# Adrenal and metabolic hormones demonstrate risk–reward trade-offs for African elephants foraging in human-dominated landscapes

**DOI:** 10.1093/conphys/coae051

**Published:** 2024-08-02

**Authors:** Sandy Oduor, Nathaniel N Gichuki, Janine L Brown, Jenna Parker, Dennis Kimata, Suzan Murray, Shifra Z Goldenberg, Maurice Schutgens, George Wittemyer

**Affiliations:** Department of Biology, University of Nairobi, PO Box 30197-00100, Nairobi, Kenya; Department of Reproductive Biology, Smithsonian Conservation Biology Institute, Front Royal, VA 22630, USA; Department of Biology, University of Nairobi, PO Box 30197-00100, Nairobi, Kenya; Center for Species Survival, Smithsonian Conservation Biology Institute, Front Royal, VA 22630, USA; Conservation Science and Wildlife Health, San Diego Zoo Wildlife Alliance, San Diego, CA 92027, USA; Department of Biology, University of Nairobi, PO Box 30197-00100, Nairobi, Kenya; Global Health Program, Smithsonian Conservation Biology Institute, Washington, DC, USA; Conservation Science and Wildlife Health, San Diego Zoo Wildlife Alliance, San Diego, CA 92027, USA; Conservation Science Department, Space for Giants, PO Box 174-10400, Nanyuki, Kenya; Department of Fish, Wildlife, and Conservation Biology, Colorado State University, Fort Collins, CO, USA; Save the Elephants, P. O. Box, 54667 - 00200, Nairobi, Kenya

**Keywords:** Faecal glucocorticoid metabolites, human modification index, human–elephant conflict, land use, thyroid hormones, Abbreviations: fGCM, faecal glucocorticoid metabolite, fT3, faecal thyroid metabolite, GC, glucocorticoid, GVIF, generalized variation inflation factor, NDVI, normalized difference vegetation index

## Abstract

A key driver of the African savannah elephant population decline is the loss of habitat and associated human–elephant conflict. Elephant physiological responses to these pressures, however, are largely unknown. To address this knowledge gap, we evaluated faecal glucocorticoid metabolite (fGCM) concentrations as an indicator of adrenal activity and faecal thyroid metabolite (fT3) concentrations as an indicator of metabolic activity in relation to land use, livestock density, and human landscape modification, while controlling for the effects of seasonality and primary productivity (measured using the normalized difference vegetation index). Our best-fit model found that fGCM concentrations to be elevated during the dry season, in areas with higher human modification index values, and those with more agropastoral activities and livestock. There was also a negative relationship between primary productivity and fGCM concentrations. We found fT3 concentrations to be higher during the wet season, in agropastoral landscapes, in locations with higher human activity, and in areas with no livestock. This study highlights how elephants balance nutritional rewards and risks in foraging decisions when using human-dominated landscapes, results that can serve to better interpret elephant behaviour at the human–wildlife interface and contribute to more insightful conservation strategies.

## Teaser

Understanding the adrenal and metabolic state of wild African elephants in human dominated areas is important in evaluating the risk-reward tradeoffs that elephants face in their navigation of a complex landscape, including protected and unprotected areas with different resource stressors. We found that both glucocorticoid and thyroid hormones were affected by land use and human activities.

## Introduction

Biodiversity in Africa is increasingly threatened by habitat loss and fragmentation, climate change, overexploitation of natural resources, invasive species, and pollution (Tilman *et al*., 2017; Leisher *et al*., 2022), resulting in many of the continent’s species being at risk of extinction (Maxwell *et al*., 2016). Protected areas alone are inadequate to safeguard the diversity of species and the ranges they require, particularly in the face of uncertainty brought by climate change (Western *et al*., 2020). The importance of multi-use and human-occupied landscapes such as community conservancies, communal pastoral lands, and private ranches is increasingly recognized as critical to conservation efforts as they harbour important habitats and migratory corridors for wildlife populations (Kiffner *et al*., 2020; Frank, 2023).

Identifying drivers of use and avoidance in human-occupied areas is critical for understanding the habitat characteristics that drive animal attraction to unprotected areas (Abram *et al*., 2022; Riggio *et al*., 2022). Population monitoring through cameras or tracking collars provides valuable information on distribution and resource selection (Gaynor *et al*., 2018; Bastille-Rousseau *et al*., 2020) but has limited ability to discern the costs and benefits of using particular areas. Physiological data derived from animals navigating varied landscapes provide opportunities to discern the costs and benefits of these uses.

Measures of endocrine functionality are an essential approach to understanding animals’ ability to cope with or respond to anthropogenic threats (Bradshaw, 2007; Denver *et al*., 2009; Dantzer *et al*., 2014). An immediate physiological response to an external stressor is partly regulated by the hypothalamic–pituitary–adrenal axis and involves the release of glucocorticoid (GC) hormones from the adrenal glands (Romero, 2004). Under normal circumstances, GCs play an important role in regulating energy and maintaining homeostasis in response to adverse events or environments (MacDougall-Shackleton *et al*., 2019). However, prolonged elevations in blood GC concentrations can lead to negative health consequences, including suppressed immune function, increased disease susceptibility, inhibited reproduction, and decreased growth (Sapolsky *et al*., 2000; Busch and Hayward, 2009; Romero and Wingfield, 2016), all of which affect individual fitness and potentially population viability.

Other endocrine biomarkers include thyroid hormones, which function to increase basal metabolic rate, make more glucose available to cells, stimulate protein synthesis, increase lipid metabolism and stimulate cardiac and neural functions (Pasciu *et al*., 2022, 2024). Thyroid hormones are activated by the hypothalamic–pituitary–thyroid axis, resulting in the production of tetraiodothyronine (T4) and triiodothyronine (T3) from thyroid follicles (Behringer *et al*., 2018). Both GC and thyroid hormones are regulators of metabolic pathways. For instance, GC hormones are tightly linked to metabolism by converting stored energy into glucose to respond to challenges (Busch and Hayward, 2009; Little and Seebacher, 2024). As a result, GC concentrations correlate with energy expenditure (Sapolsky *et al*., 2000; Romero and Wingfield, 2016) and increase during routine energetic demands for high-energy life stages, including reproduction (Brown and Lehnhardt, 1995; Fanson *et al*., 2014). Thyroid hormones (both T4 and T3) are responsive to nutritional fluctuations and can lower metabolism to conserve energy during nutritional emergencies (Flier *et al*., 2000; Wasser *et al*., 2010). In elephants, GC and T3 hormones can be measured non-invasively as metabolites in faeces [i.e. faecal glucocorticoid metabolite (fGCM) and faecal thyroid metabolite (fT3)] and reflect the hormonal state about 36 hours preceding defecation (Wasser *et al*., 2000), making these valuable tools for assessing stress and metabolic status. Hence, measures of both fGCM and fT3 would be useful for discerning nutritional from non-nutritional stressors and providing greater insights into different coping strategies in response to environmental challenges.

African savannah elephants (*Loxodonta africana*) are an ideal species for examining how animals physiologically adjust to human activity, landscape modification, climate change and associated threats (Madliger *et al*., 2018). Elephants inhabit areas both within and outside of protected areas, including those undergoing rapid anthropogenic change (Wall *et al*., 2021), providing opportunities to examine physiological responses to a myriad of human-induced stressors. In this study, we examined fGCM and fT3 concentrations in African savannah elephants across the mixed-use Laikipia–Samburu ecosystem of northern Kenya (Ihwagi, 2019). The area has been experiencing increased anthropogenic pressure exacerbated by increased human population growth, increased livestock densities and associated overgrazing and increased sedentarization of pastoral lands (Letai and Lind, 2013), all of which are altering ecological processes in the ecosystem.

We tested predictions that elephants would have higher fGCM concentrations in agropastoral landscapes, areas with higher livestock abundance, and those with greater human modification due to the increased likelihood of negative human interactions. We also tested predictions that elephants would have higher fGCM concentrations during the dry season with lower food availability and quality. For fT3, we predicted that elephants would have higher concentrations in agropastoral landscapes where agricultural resources of high nutritional value are available, and during the wet season when vegetation productivity is higher. These findings could provide insight into how human activities impact physiological function in elephants and, ultimately, fitness and survival.

## Materials and Methods

### Study sites

The study was conducted within the Laikipia–Samburu ecosystem (0.4°S to 2°N, 36°E to 38.5°E), an expansive 33 817 km^2^ semi-arid rangeland in the northern part of Kenya, defined mainly by the historical range of the elephant population that uses it (Thouless, 1995; Ihwagi *et al*., 2015). The study area hosts the second largest population of African savannah elephants in Kenya, with a population of about 7475 individuals (Waweru *et al*., 2021). The ecosystem is semi-arid, comprising a wide range of habitats from dense thorny woodlands (dominated by *Commiphora schimperi*, *C. incisa* and *C. africana*) in the north, riverine vegetation of semi-arid scrub around the Ewaso Nyiro River, to a more mesic, deciduous highland in the south (Thouless, 1995; Duporge *et al*., 2022). Rainfall is mainly bimodal, with an annual gradient decreasing from 400–1200 mm in the south to 250–500 mm in the north, except around the Mathews Range, where rainfall can reach up to 1250 mm (Kimiti *et al*., 2017).

**Figure 1:**
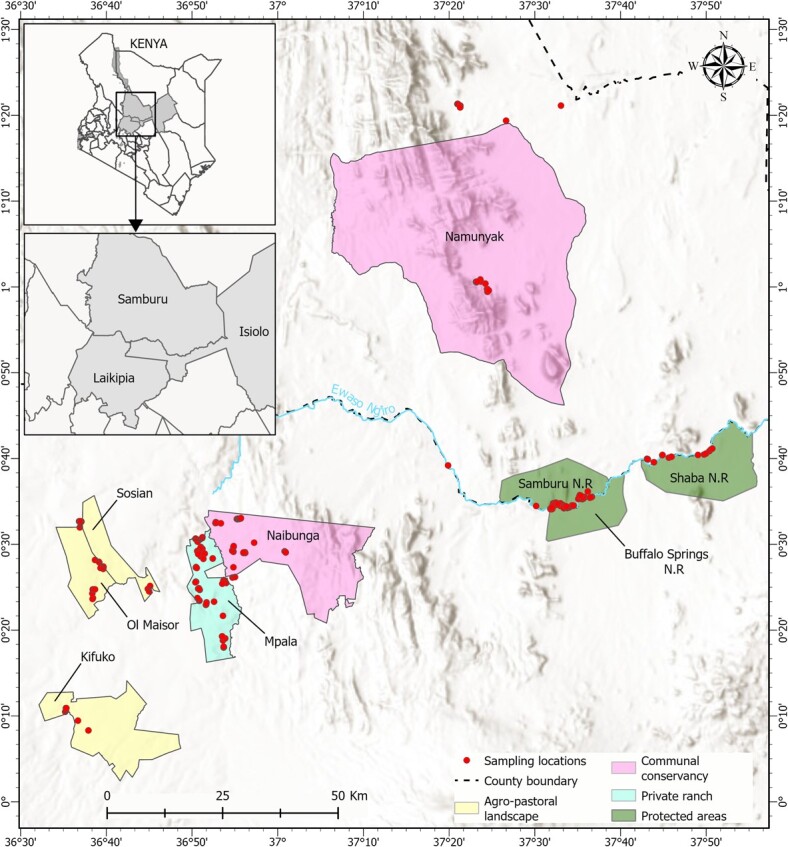
Map of the study area showing different land use types including national reserves, private ranches, community conservancies and agropastoral landscapes.

The study was conducted across four main land use types: (i) national reserves, (ii) community conservancies, (iii) private ranches and (iv) agropastoral landscapes (Fig. 1). National reserves are protected areas designated by the government for the conservation and management of wildlife by county governments, represented in this study by Samburu National Reserve, Buffalo Springs National Reserve, and Shaba National Reserve, which together cover an area of about 533 km^2^ (Ihwagi *et al*., 2015). The vegetation of the reserves is characterized by *Vachellia*—*Commiphora* semi-arid scrub woodland and *Vachellia* wooded grassland (Wittemyer, 2001). Community conservancies are communally owned lands (consisting of mixed-use landscapes containing both wildlife and livestock) that are managed by a board of community representatives with the aim of conserving wildlife, managing rangelands, ecotourism, and other livelihood-related activities (Mkutu, 2020). Community conservancies in this study were represented by Naibung’a Wildlife Conservancy and Namunyak Wildlife Conservancy, which together cover an area of about 3900 km^2^. The vegetation of Naibung’a Wildlife Conservancy is characterized by woody vegetation, with *Vachellia etbaica*, *Vachellia brevispica*, *Vachellia tortilis*, *Vachellia mellifera* and *Vachellia drepanolobium* being the most dominant species (Young *et al*., 1995). Namunyak Wildlife Conservancy, on the other hand, is an ecotone between semi-arid woodland (characterized by *V. tortilis*, *Vachellia seyal* and *C. africana*) and evergreen forest within Mathews Range characterized by *Vachellia*, *Commiphora*, *Cordia* and *Newtonia* species (Egna *et al*., 2020). Private ranches are privately owned landholdings where land is leased to private individuals for the purpose of ranching, wildlife conservation and ecotourism (Sundaresan and Riginos, 2010). Private ranches in this study were represented by Mpala Ranch, a 200 km^2^ landholding managed for both wildlife conservation and livestock production. Training by the British Army Training Unit in Kenya (BATUK) also occurs twice a year (Awuor, 2015) on Mpala Ranch. Mpala Ranch is characterized by woody vegetation, with *Vachellia brevispica*, *V. mellifera*, *V. etbaica* and *V. drepanolobium* being the most dominant species (Pringle *et al*., 2016). Finally, agropastoral landscapes (mainly occurring in the west of Laikipia) are land use types where human cultivation borders fragmented habitats due to the spatially chaotic juxtaposition of natural habitats and scattered smallholder farms, shaped by land policies during the colonial and post-colonial periods in Kenya (Evans and Adams, 2016). Agropastoral landscapes in this study were represented by Ol Maisor Ranch, Kifuko Ranch and Sosian Ranch which cover a combined area of 258 km^2^. Agropastoral landscapes consist of small-holder farms ranging between 0.5 and 2 ha. The landscapes are adjacent to small natural areas that can act as a refuge for elephants during the day when they are not crop raiding. The vegetation is characterized by open woodland dominated by *Vachellia drepanolobium* (Graham *et al*., 2009).

### Faecal sampling

Fresh faecal samples were collected across the four main land use types during the dry and wet seasons. To avoid autocorrelation, each family group was visited no more than once with samples collected from each individual no more than once. Dry and wet seasons were defined as described by Wittemyer *et al*. (2005). The dry season began 30 days after a period of no rain, while the wet season began after 1 week of 15 mm of rain or more. In the dry season, faecal samples were collected between 18 March 2022–28 July 2022 and 16 October 2022–31 October 2022. In the wet season, faecal samples were collected between 11 January 2023–31 January 2023 after the October–December short rainy season and between 29 April 2023–7 June 2023 after the March–May long rainy season. A total of 294 elephant faecal samples were collected during the dry season and 260 samples during the wet season (Table 1).

**Table 1:** Summary of elephant faecal samples collected by land use category during the dry and wet seasons within the Laikipia–Samburu ecosystem, Kenya

	Season	
Land use type	Dry	Wet
Community conservancy	95	110
Private ranch	61	60
National reserve	92	58
Agropastoral landscape	46	32
Total	294	260

To avoid pseudo-replication, elephants were identified by catalogue recognition files, one used by Save the Elephants in the national reserve (Wittemyer, 2001) and another used at Mpala Ranch (Ochieng, 2015). In cases where individuals could not be identified, photographs of each elephant’s ears and tusks were taken and added to the database. Elephants were located by driving on existing roads and off roads where navigation was possible. For each sample, the time of defecation, time of collection, GPS location, age group [juvenile = 0–8 years; sub-adult = 9–17 years; adult = ≥ 18 years, based on known ages or established ageing criteria (Moss, 1996)] as a measure of life-history stage and social status, and land use type (national reserve, community conservancy, private ranch and agropastoral landscape) were recorded. In the event that an individual could not be identified from the catalogue recognition file (accounting for 27% of the total samples collected during the study period), we collected fresh dung samples and measured the circumference of the dung bolus and assigned age categories as follows; juveniles (5–6 cm), sub-adult (7–10 cm) and adult (> 11 cm) as described by Morrison *et al*. (2005).

Approximately 200 g of dung from several boluses of a single defecation event was placed in a whirl-pack bag and labelled based on the subject ID, date and location. It was then placed into a cool box with ice packs in the field before being transferred to a −20°C freezer within eight hours. Livestock density was assessed visually within a 500-m radius of the faecal sample collection point. Livestock density was recorded as: no livestock = 0 individuals, low = 1–50 individuals and high ≥51. The livestock counted within a 500-m radius of the sample collection included cattle, shoats (i.e. sheep and goats) and camels.

### Faecal sample processing and analyses

Hormones were extracted using an established wet-weight vortexing method (Edwards *et al*., 2014). All extractions and analyses were carried out at the Endocrinology Laboratory, Mpala Research Centre. In summary, samples were thawed, thoroughly mixed and 0.5 g (± 0.02) extracted by vortexing in 5 ml of 90% methanol in 16 × 125 mm glass tubes for 30 minutes followed by centrifuging at 2500 rpm for 20 minutes. The resulting supernatants were decanted into another set of 16 × 125 mm tubes and dried under air in a warm water bath, reconstituted with 1 mL of assay buffer (Cat. No. X065, Arbor Assays, Ann Arbor, MI USA), sonicated until completely resuspended and then frozen at -20°C until analysis. Concentrations of fGCM and fT3 were measured by enzyme immunoassay (EIA) (DetectX® Corticosterone EIA K014, Arbor Assays, Ann Arbor, MI and DetectX® Triiodothyronine EIA K056, Arbor Assays, Ann Arbor, MI) as described by Oduor *et al*. (2020) and Szott *et al*. (2020), respectively. The EIA sensitivity for both corticosterone and T3 was 0.90 pg/well (at 90% binding). Intra- and inter-assay coefficients of variation for the corticosterone EIA were 7.6% and 9.8%, respectively, and 8.7% and 10.4%, respectively, for the T3 EIA. Serial dilutions of faecal extracts in assay buffer were parallel to the standard curve for corticosterone (*R*^2^ = 0.9692, *y* = 0.98*x* + 18.57; *P* < 0.05) and T3 (*R*^2^ = 0.9362, *y* = 1.1*x*−26.67; *P* < 0.05). Faecal extracts were diluted 1:4–1:16 and 1:20–1:90 for analysis of GC and T3 metabolites, respectively.

### Human modification index

To characterize differences in human presence and impact across the land use areas, we used the Human Modification Index (HMI)—a tool for capturing the multidimensional, changing influence of humans on land. The HMI was calculated elsewhere as a continuous scale of increasing human pressure from 0 to 1 (Gustafson and Parker, 1992). Human pressure on the landscape that directly or indirectly alters natural systems were quantified by aggregating the 13 stressors described by Kennedy *et al*. (2019) to a cumulative score using a ‘fuzzy algebraic sum’ (Bonham-Carter, 1994; Perkl, 2017). The index was scaled from 0.00 indicating no human impact to 1.00 indicating high human impact. The cumulative human modification map generated was processed in a code editor (JavaScript) interface from Google Earth Engine (GEE) provided at (Global Human Modification) with a resolution of 1 km^2^ (Kennedy *et al*., 2019). The resulting raster image was exported to R programing for statistical analysis (R Development Core Team, 2024), where an HMI value was extracted for each sampling point across the study areas.

### Normalized difference vegetation index

Normalized difference vegetation index (NDVI) was used to characterize productivity. NDVI values were calculated for GPS locations of faecal sample collections using Moderate Resolution Imaging Spectroradiometer (MODIS) images, compiled at 16-day intervals at a 250 m resolution (MODIS_061_MOD13Q1). For every faecal sample, the intersected NDVI value (at the 250 m MODIS pixel resolution) for the overlapping time window (16-day interval) was extracted using Google Earth Engine (Crego *et al*., 2021).

### Statistical analysis

**Figure 2:**
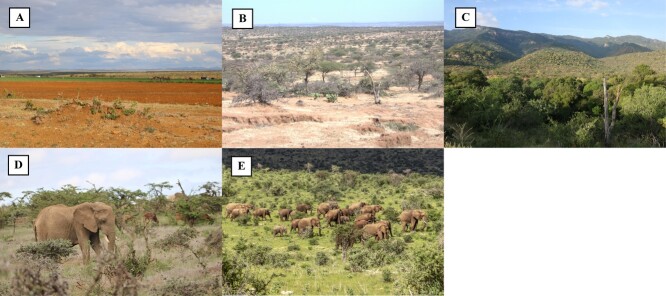
An image of land use types represented by different land use categories including (**A**) agropastoral landscape—cultivated farmlands are adjacent to the natural vegetation which appears in the background of the image where elephants take refuge when they are not crop raiding; (**B**) community conservancy (Naibung’a)—open woodland dominated by *Vachellia* spp., with mostly bare ground due to overgrazing. The area also has a high density of the invasive *Opuntia* species; (**C**) community conservancy (Namunyak)—characterized by dense thorny woody vegetation with Mathew’s Range Mountains in the background; (**D**) private ranch—open woodland vegetation with lower levels of overgrazing and (**E**) national reserve—open grassland with scattered shrubland in the background.

We constructed multiple linear regression models for which the response variable was the log-transformed (for normality) fGCM or fT3 concentrations. In both sets of models, we included the effect of: (i) land use categories as described above (four categories); (ii) land use location (a categorical variable with six categories for each sampled location as shown in Fig. 1 and described below); (iii) HMI; (iv) livestock density; (v) season; (vi) NDVI; and (vii) age group on fGCM and fT3 concentrations in African elephants. We included both linear and quadratic functions for NDVI and HMI in the model to determine which better explained variation in the data. As described above, the four land use categories were based on land ownership systems by different stakeholders and law enforcement authorities: (i) private ranch; (ii) community conservancy; (iii) national reserve and (iv) agropastoral landscapes (Supplementary Table 1). To account for the spatial contiguity and heterogeneity of human activities across the locations, different locations within the study area were merged into six categories as follows: (i) agropastoral site with agriculture (Ol Maisor); (ii) agropastoral site without agriculture (Sosian-Kifuko); (iii) community conservancy with pastoralism only (Namunyak Wildlife Conservancy); (iv) community conservancy with mixed use (Naibung’a Wildlife Conservancy); (v) national reserves (Samburu National Reserve, Buffalo Springs National Reserve and Shaba National Reserve); and (vi) private ranch (Mpala Ranch). The agropastoral site with agriculture had both livestock production and farming taking place at the time of the study. Agropastoral sites without agriculture were sites where both livestock keeping and agricultural practices have occurred, but only livestock keeping was taking place at the time of the study. Community conservancy with pastoralism only is community owned land where pastoralism occurs. Community conservancy with mixed use is community owned land where both pastoralism and subsistence farming on a small scale occurs (Fig. 2).

To derive the most parsimonious model, we selected the top model for each hormone (Supplementary Table 2 for fGCM and Supplementary Table 3 for fT3) based on Akaike’s Information Criterion adjusted for small sample sizes (Burnham and Anderson, 2002). We compared our best-fit model to the null model. The residuals of the top models were visually assessed for normality and heteroskedasticity. Multicollinearity in the predictor variables for the best models were assessed in the CAR package (Fox and Weisberg, 2018) using a generalized variation inflation factor (GVIF) analysis (Fox and Weisberg, 2018). GVIF values corrected for the degrees of freedom (i.e. ${\mathrm{GVIF}}^{\frac{1}{2 df}})$ were found to be lower than the required threshold of 3 as described by (Zuur *et al*., 2010). All statistical analyses were performed in the statistical program R version 4.4.0 (R Development Core Team, 2024). Mean data are expressed as ± standard deviation (SD).

## Results

### Descriptive results

fGCM concentrations (*n* = 554) averaged (± SD) 8.30 ± 9.12 ng/g and ranged from 2.03 to 96.22 ng/g. fT3 concentrations (*n* = 554) averaged 187.79 ± 248.28 ng/g and ranged from 11.75 to 2343.41 ng/g. fGCM concentrations differed across seasons, with the dry season having significantly higher fGCM concentrations 9.28 ± 10.46 ng/g compared to the wet season 7.18 ± 7.16 ng/g [*t*(520.30) = 2.78 (*P* < 0.05)]. fT3 concentrations differed across seasons, with the wet season having significantly higher fT3 concentrations 231.64 ± 301.10 ng/g compared to the dry season 149.01 ± 181.63 ng/g [t(414.56) = −3.85 (*P* < 0.05)]. Mean fGCM concentrations were higher in sub-adults (8.98 ± 11.99 ng/g, *n* = 176) compared to juveniles (8.18 ± 5.80 ng/g, *n* = 116) and adults (7.89 ± 8.03 ng/g, *n* = 262). Mean fT3 concentrations were higher in adults (194.15 ± 251.44 ng/g, *n* = 262) compared to juveniles (183.31 ± 203.82 ng/g, *n* = 116) and sub-adults (181.28 ± 270.33 ng/g, *n* = 176).

### Predictors of fGCM concentrations

**Figure 3:**
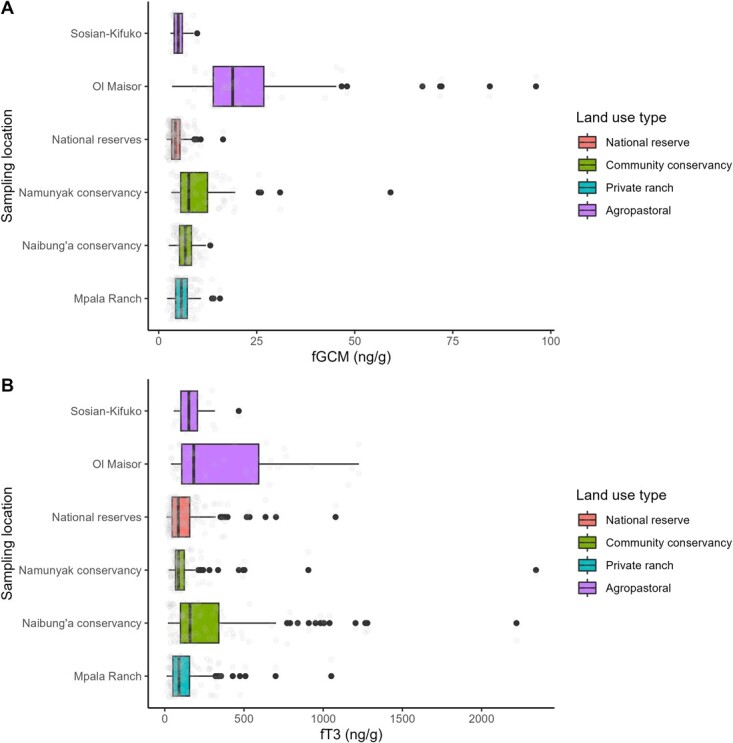
Influence of land use on fGCM and fT3 concentrations. Box plots of (**A**) fGCM and (**B**) fT3 concentrations in African elephants in different locations across land use systems. Each box denotes the interquartile range and mean as a black line within the box. The grey dots represent the fitted data points while the black points above the box plots represent outliers.

The top model for fGCM concentrations consisted of sampling location, HMI, number of livestock within a 500-m radius, season, NDVI, and age group [*F*(12, 541) = 54.42, *p* = < 0.05, *R*^2^ = 0.54] (Supplementary Table 2). fGCM concentrations differed across locations. Relative to the reference location of Mpala Ranch, the agropastoral site with agriculture had the highest concentrations (Ol Maisor [coefficient estimate = 1.13, 95% confidence interval (CI) = 0.91–1.36] followed by community conservancy with pastoralism only (Namunyak Conservancy (0.47, 95% CI = 0.31–0.63), community conservancy with mixed-use (Naibung’a Conservancy (0.04, 95% CI = −0.11 to −0.18), agropastoral site without agriculture (Sosian-Kifuko landscape (−0.23, 95% CI = −0.44 to −0.02) and the lowest concentrations in Samburu and Buffalo Springs National Reserves (−0.28, 95% CI = −0.38 to −0.17) (Fig. 3, Table 2).

**Table 2:** Model estimates from the best-fitting model, showing variation of fGCM concentrations in African elephants according to different predictor variables. The reference category for location is Mpala Ranch, the reference category for livestock within a 500-m radius is no livestock and the reference category for age is Juvenile

Model: log(fGCM) ~ Location + HMI + Season + Livestock within 500 m radius + NDVI + Age group					
Predictors	Estimate	Std. Error	CI	Statistic	P
Intercept	2.05	0.12	1.81 to 2.28	9.42	**<0.001**
Location [Naibung’a conservancy]	0.04	0.07	−0.11 to −0.18	−0.22	0.599
Location [Namunyak conservancy]	0.47	0.08	0.31 to 0.63	5.49	**<0.001**
Location [National reserves]	−0.28	0.05	−0.38 to −0.17	−3.63	**<0.001**
Location [Ol Maisor]	1.13	0.12	0.91 to 1.36	10.81	**<0.001**
Location [Sosian-Kifuko]	−0.23	0.11	−0.44 to −0.02	−2.35	**0.032**
HMI	0.66	0.28	0.10 to 1.22	3.04	**0.021**
Season [Wet Season]	−0.09	0.04	−0.17 to −0.02	−0.74	**0.018**
Livestock within 500 m radius [Low Livestock]	0.18	0.06	0.06 to 0.30	3.08	**0.003**
Livestock within 500 m radius [high livestock]	0.05	0.06	−0.07 to 0.18	0.76	0.417
NDVI	−1.96	0.40	−2.75 to −1.17	−5.03	**<0.001**
Age group [sub-adult]	−0.02	0.05	−0.12 to 0.07	0.25	0.622
Age group [adult]	0.06	0.05	−0.03 to 0.15	1.52	0.198
Observations	554				
R^2^/R^2^ adjusted	0.55/0.54				

Sampling locations with low (0.18 relative to the reference category of no livestock, 95% CI = 0.06–0.30) and high (0.05, 95% CI = −0.07 to −0.02) livestock density were estimated to have higher fGCM concentrations relative to those with no livestock. fGCM concentrations were also estimated to be lower during the wet season than the dry season (−0.09 relative to the reference category of dry season, 95% CI = −0.17 to −0.02). fGCM concentrations were positively correlated with HMI (0.66, 95% CI = 0.10–1.22) (Fig. 3) and negatively correlated with NDVI (−1.96, 95% CI = −2.75 to −1.17). fGCM concentrations estimated for adults (0.06, 95% CI = −0.03 to −0.15) and sub-adults (−0.02, 95% CI = −0.12 to 0.07) did not significantly differ relative to juveniles.

### Predictors of fT3 concentrations

**Figure 4:**
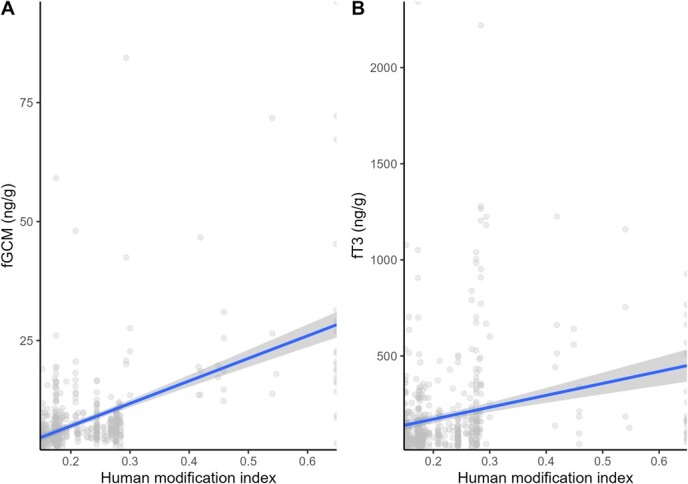
Linear regression plots with 95% confidence intervals (grey areas) showing the relationship between (**A**) fGCM and (**B**) fT3 concentrations and the human modification index with fitted data points in grey.

The top model for fT3 concentrations consisted of sampling location, HMI, number of livestock within a 500-m radius and season [*F*(9, 544) = 17.19, *P* = < 0.05, *R*^2^ = 0.21] (Supplementary Table 3). fT3 concentrations differed across locations with the agropastoral site with agriculture having the highest estimated concentrations (Ol Maisor (0.88, 95% CI = 0.45–1.32), followed by community conservancy with mixed-use (Naibung’a Conservancy (0.68, 95% CI = 0.40–0.96), agropastoral site without agriculture (Sosian-Kifuko (0.54, 95% CI 0.14–0.95), community conservancy with pastoralism only (Namunyak Conservancy (0.19, 95% CI −0.08 to −0.45) and the lowest concentrations in national reserves (0.04, 95% CI −0.17 to −0.24) (Fig. 2, Table 3). fT3 concentrations differed across seasons with the wet season having greater fT3 concentrations relative to the dry season (0.47, 95% CI 0.36–0.66). Additionally, fT3 concentrations were positively correlated with HMI (1.09, 95% CI 0.00–2.18) (Fig. 4). Sampling locations with no livestock had higher fT3 concentrations relative to areas with either low (−0.39, 95% CI −0.63 to −0.15) and high (−0.13, 95% CI −0.38 to −0.12) livestock density, although confidence intervals for the latter estimate overlapped zero.

**Table 3:** Model estimates from the best-fitting model, showing variation of fT3 concentrations in African elephants according to different predictor variables. The reference category for location is Mpala Ranch, and the reference category for livestock within a 500-m radius is no livestock

Model: log(fT3) ~ Location + HMI + Season + Livestock within 500 m radius					
Predictors	Estimate	Std. Error	CI	Statistic	*P*
Intercept	4.16	0.14	3.89 to 4.43	6.61	**<0.001**
Location [Naibung’a conservancy]	0.68	0.14	0.40 to 0.96	3.98	**<0.001**
Location [Namunyak conservancy]	0.19	0.14	−0.08 to 0.45	0.24	0.173
Location [National reserves]	0.04	0.10	−0.17 to 0.24	0.86	0.715
Location [Ol Maisor]	0.88	0.22	0.45 to 1.32	3.88	**<0.001**
Location [Sosian-Kifuko]	0.54	0.21	0.14 to 0.95	1.09	**0.009**
HMI	1.09	0.56	0.00 to 2.18	1.77	**0.050**
Season [wet season]	0.47	0.08	0.31 to 0.62	6.27	**<0.001**
Livestock within 500 m radius [low livestock]	−0.39	0.12	−0.63 to −0.15	−1.74	**0.001**
Livestock within 500 m radius [high livestock]	−0.13	0.13	−0.38 to 0.12	0.21	0.301
Observations	554				
R^2^/R^2^ adjusted	0.22/0.21				

## Discussion

We investigated the effects of land use on the physiological stress response (as measured by fGCM concentrations) and metabolic activity (as measured by fT3 concentrations) of African elephants within the Laikipia–Samburu ecosystem, a mixed-use system representing land uses with varying levels of human impact (Duporge *et al*., 2022). We found that elephants within agropastoral sites with agriculture (Ol Maisor), where crop raiding and associated retaliation can occur, had both higher fGCM and higher fT3 concentrations compared to elephants within national reserves, community conservancies or private ranches. We also found higher fGCM concentrations during the dry season and higher fT3 concentrations during the wet season, whereby fluctuations were correlated with changes in NDVI (fGCM concentrations were negatively correlated with NDVI). We explored several metrics of human presence on the landscape, generally finding higher fGCM in areas with greater signs of human activity (high HMI and low to high livestock density), which were similarly positively correlated with fT3 concentrations. However, we did not find a strong relationship between fGCM concentrations and our demographic metric of age (representing life history stage and social status).

Our results depicting the positive correlation between fGCM and fT3 and anthropogenic land use are largely aligned with the results of other studies on elephants, though few have explored hormone concentrations across such a diverse, predominantly unprotected landscape mosaic. Higher fGCM concentrations have been found among African elephants were found outside of protected areas in Etosha National Park in Namibia (Hunninck *et al*., 2017), conservation areas where some human activities are allowed within the Amboseli–Mara ecosystem of Kenya (Ahlering *et al*., 2013), and outside the boundary of the Serengeti National Park in Tanzania (Tingvold *et al*., 2013). Similarly, in Asian elephants (*Elephas maximus*), Tang *et al*. (2020) observed higher fGCM concentrations among individuals who occupied disturbed sites near tea plantations in China compared to those who were in undisturbed sites within a national park. Predators such as African lions (*Panthera leo*) have shown a similar trend, with higher fGCM concentrations observed among individuals found in human-settled buffer zones compared to those within community conservation areas in the South Rift region of Kenya (Creel *et al.*, 2013). Our results indicated that elephant stress levels were significantly lower in the national reserves than in all other land use areas assessed, indicating these areas serve as important refuges from human influence. Previous work highlighted the generally calm nature of the elephants using these protected areas, even during periods of high poaching in the ecosystem (Goldenberg *et al*., 2017). The reserve elephants are habituated to vehicles and tourist presence, which may drive the reduced stress levels reported here.

Areas within agropastoral landscapes are predominantly high-productivity areas, including agriculture, with many human settlements and strong human influences, which likely underpinned the greater stress in elephants using the areas as reflected by fGCM concentrations. We observed a strong positive correlation between fGCM concentrations and HMI at dung sample sites, which indicates that proximity to human activities activates the physiological stress response of African elephants. In addition, elephant crop raiding is usually rampant within agropastoral landscapes in the Laikipia–Samburu ecosystem, invoking retaliatory attacks on elephants in response to crop losses (Graham *et al*., 2010). This is potentially another reason why higher fGCM concentrations were observed in agropastoral landscapes compared to other areas without agricultural developments.

In addition to fGCM differences, we also observed higher concentrations of fT3 in an agropastoral site with agriculture (Ol Maisor) compared to other land use types. This pattern is consistent with studies from other wildlife species which have observed higher fT3 concentrations in relation to higher energy acquisition in mantled howler monkeys (*Alouatta palliata*) (i.e., fruits and young leaves) (Dias *et al*., 2017), and during peak harvest activity in maned wolves (*Chrysocyon brachyurus*), presumably due to the use of those human-generated resources (Vynne *et al*., 2014). The high fT3 concentrations in elephants in agropastoral landscapes with agriculture likely reflect a higher caloric intake relative to other land use types, potentially indicating the benefits of agricultural use by elephants (Chiyo *et al*., 2011).

Though we found higher fGCM and lower fT3 concentrations during the dry season as predicted, it is possible our results were structured by an extreme drought in northern Kenya during the study. The reported hormonal relationships might change or be less evident in non-drought years. In other studies, this relationship has also been found. Higher fGCM concentrations were found among African elephants during the dry season in Kruger National Park in South Africa (Viljoen *et al*., 2008) and Asian elephants in Thailand (Norkaew *et al*., 2019) and southern India when their body condition scores decreased with forage availability (Pokharel *et al*., 2017). In several zoos in the USA, Mondol *et al*. (2020) found higher fGCM and lower fT3 concentrations in response to both physiological and nutritional challenges.

Like seasonality, NDVI in savanna systems can be a useful measure of variability in forage quality, with greenness indicating higher net primary productivity. Other studies have found a negative correlation between fGCM concentrations and NDVI in free-ranging African elephants (Oduor *et al*., 2020; Parker *et al*., 2022). Although the study found the influence of season as categorized coarsely by rainfall on fT3 concentrations, our most parsimonious model for fT3 concentrations did not include NDVI as one of the covariates explaining variation in our data. This was surprising, given that we assumed NDVI as an index of primary productivity would correlate with the availability and nutritional quality of forage. The wide area assessed, with different ecological communities and human activities, likely impacts elephant diets across systems, which may reduce any relationship between NDVI and fT3. For instance, in one of the community conservancies (Naibung’a), elephants have been feeding on invasive *Opuntia* spp., which is perceived as a major threat to the ecological integrity of the area and appears to be driving human–elephant conflict (Strum *et al.*, 2015) by attracting elephants to areas near people where *Opuntia* colonization is common. Our results indicate that high fT3 concentrations in Naibung’a are second only to the agriculture area in the study system despite being an area with generally lower NDVI values. Presumably, this is because Naibung’a has high *Opuntia* density, likely indicating that elephants derive nutritional value from this invasive plant (Fig. 2B). In other ungulate studies, only a partial influence of fT3 concentrations on NDVI has been observed. For instance, Hunninck *et al*. (2020) found that fT3 concentrations in impalas (*Aepyceros melampus*) only had an influence on NDVI within the Serengeti ecosystem when ambient temperature was accounted for in the analysis. Nevertheless, our findings illustrate the role that endocrine biomarkers play in understanding how African elephants adjust to energetic demands in the face of environmental challenges, which will be critical for the conservation of species in the face of human-driven ecological and climate change.

Although a decline in biodiversity has been partly associated with increasing livestock numbers (Ogutu *et al*., 2016), to our knowledge, no study has examined how wildlife physiologically adjusts to different levels of livestock densities in multi-use landscapes. Our study found that elephants sampled in areas with no livestock had significantly lower fGCM concentrations and higher fT3 concentrations relative to areas with low or high livestock density. It is important to note that measures of fGCM in elephants reflect the hormonal state about 36 hours prior to sampling (Wasser *et al*., 2000). The point count of livestock when samples were collected may not reflect the same level of exposure experienced by the elephants 36 hours prior, as both livestock and elephants are highly mobile. However, land use is heterogeneous across the landscape, and uncertainty may be primarily related to discerning high- versus low-density livestock areas, while presence versus absence may better reflect the circumstances experienced by elephants. It is notable that indirect ecological benefits of livestock presence on wild herbivores have been observed. Livestock can facilitate the growth of grassland during the dry season and help maintain habitat heterogeneity (Odadi *et al*., 2011; Young *et al*., 2018), which presumably could result in higher fT3 where livestock densities are greater. However, we found the opposite in this study. In a multi-use landscape where resources are shared by both livestock and wild herbivores, livestock presence can influence habitat use by wildlife (Connolly *et al*., 2021; Wells *et al*., 2022). Indeed, Kinnaird and O'Brien (2012) noted that wildlife occupancy was reduced with higher stocking levels. Studies have also observed strong avoidance behaviour of African elephants in areas recently grazed by cattle in the Greater Mara ecosystem (Herrik *et al*., 2023), representing risk avoidance behaviour in human-dominated landscapes (Graham *et al*., 2009), which may influence nutritional access. Further, pastoralists and elephants can come into conflict over water during dry seasons (Wittemyer *et al*., 2017). Our study suggests that elephants respond physiologically to these interactions.

## Conclusions

This study illustrates the importance of using physiological measures to gain insight into the physiological response of wildlife to anthropogenic pressures and how they maximize energy acquisition under environmental challenges. An overactive stress response has been linked to lower individual fitness through reductions in energy acquisition or utilization, compromised immune function and endocrine dysfunction (Cooke *et al*., 2013; Madliger and Love, 2015). Metabolic stress responses, on the other hand, have been linked to energy availability in relation to metabolic demand, reproduction, growth and maintenance (Behringer *et al*., 2018). Results from our study illustrate the role of conservation areas in cushioning wildlife against anthropogenic pressures such as agricultural land use change, urbanization, and increased competition with livestock. This was evident by the lower fGCM concentrations within the national reserves compared to other land use categories. Our study suggests that dry seasons and drought, crop raiding in agricultural areas, increased human modification of landscapes and livestock density activate adrenal and metabolic responses in African savannah elephants. The drought during the study, which resulted in the deaths of more than 70 elephants within the Laikipia–Samburu ecosystem (WRTI, 2022), likely influenced fGCM and fT3 concentrations. In particular, the very low fT3 concentrations recorded in protected areas may be related to the extreme drought hitting that region of the study ecosystem. Long-term monitoring in the ecosystem (Wittemyer *et al*., 2021) recorded drought-induced mortality in juveniles and older adults. As such, the fT3 levels recorded in this study for the national reserves can serve as a threshold level for mortality-inducing nutritional stress.

This study highlights the trade-offs elephants experience when balancing the danger of using human-dominated areas with the attraction to higher nutritional resources in those same areas. Elephants in human-dominated areas showed not only signs of better nutrition but also higher levels of stress. This underscores the importance of these areas and the costs of using them for wildlife and provides a useful metric by which to quantify the trade-offs of such areas for wildlife. As development increases across Africa, we expect elephants will increasingly have to navigate land use mosaics. Monitoring physiological change can inform our understanding of the trade-offs experienced by elephants across those landscapes. To protect most of Africa’s wildlife, increasing focus on the conservation of animals outside protected areas that experience additional challenges to physiological well-being is necessary. Endocrine monitoring can help identify the different challenges animals face in such systems.

## Supplementary Material

Web_Material_coae051

## Data Availability

Data from this article will be shared upon request to the corresponding author.
