# Supplementary material for: Adrenal and metabolic hormones demonstrate risk–reward trade-offs for African elephants foraging in human-dominated landscapes
Source: Conserv Physiol. 2024 Aug 2;12(1):coae051. doi: 10.1093/conphys/coae051 (PMC11295215; doi:10.1093/conphys/coae051)
Supplement: Web_Material_coae051 [file web_material_coae051.zip › Supplementary list of tables.pdf]

SUPPLEMENTARY LIST OF TABLES

Supplementary Table 1: Summary of different land-use types, locations where sampling was conducted and a description of the locations.

Land use type	Location	Description
National reserve	Samburu National Reserve	Protected areas designated by the Kenyan government for the conservation and management of wildlife. No or limited human activities are allowed.
	Buffalo-Spring National Reserve	
	Shaba National Reserve	
Communal conservancy	Naibung'a Wildlife Conservancy	Land is owned by the community. Pastoralism and wildlife-based tourism are the main socioeconomic activities. Farming on a subsistence basis also occurs occasionally.
	Namunyak Wildlife Conservancy	Land is owned by the community. Pastoralism and wildlife-based tourism are the main socioeconomic activities.
Agropastoral landscapes	Ol Maisoh Ranch	Predominantly agropastoral landscapes, with agriculture and livestock activities taking place at the time of the study.
	Sosian-Kifuko	Predominantly agropastoral landscapes but with only livestock production during the study.
Private ranch	Mpala Ranch	Land is leased to private individuals for the purpose of ranching and wildlife conservation. Military ballistic training also occurs twice a year.

Supplementary Table 2: Model selection based on lowest AICc value on the factors influencing fGCM concentrations in African elephants. The model consisting of location, HMI, season, livestock within a 500 m radius, NDVI and age group had the best fit (in bold).

Model	K	Model Likelihood	AICc	ΔAICc	Weight	R^2	Adj. R^2
**fGCM ~ Location + HMI + Season + Livestock density + NDVI + Age group**	**14**	**-291.43**	**611.64**	**0.00**	**0.39**	**0.55**	**0.54**
fGCM ~ Location + HMI + Season + Livestock density + NDVI	12	-293.80	612.17	0.53	0.30	0.54	0.53
fGCM ~ Location + HMI^2 + Season + Livestock density + $NDVI^2$	12	-293.80	612.17	0.53	0.30	0.54	0.53
fGCM ~ Location + HMI + Season + Livestock density	11	-305.17	632.83	21.19	0.01	0.52	0.52
fGCM ~ Location + HMI + Season	9	-310.93	640.18	28.54	0.00	0.51	0.51
fGCM ~ Location + HMI	8	-313.81	643.88	32.24	0.00	0.51	0.50
fGCM ~ Location	7	-317.30	648.81	37.17	0.00	0.50	0.50
fGCM ~ Land use type + HMI + Season	7	-363.21	740.63	128.99	0.00	0.41	0.41
fGCM ~ Land use type + HMI + Season + Livestock density	9	-362.80	743.93	132.29	0.00	0.41	0.41
fGCM ~ Land use type + HMI + Season + Livestock density + NDVI	10	-362.75	745.91	134.27	0.00	0.41	0.41
fGCM ~ Land use type + HMI^2 + Season + Livestock density + $NDVI^2$	10	-362.75	745.91	134.27	0.00	0.41	0.41
fGCM ~ Land use type + HMI + Season + Livestock density + NDVI + Age group	12	-361.92	748.43	136.78	0.00	0.42	0.40
fGCM ~ Land use type + HMI	6	-373.42	758.99	147.35	0.00	0.39	0.39
fGCM ~ Land use type	5	-407.57	825.24	213.60	0.00	0.31	0.31

Supplementary Table 3: Model selection based on lowest AICc value on the factors influencing fT3 concentrations in African elephants. The model consisting of location, HMI, season and livestock within a 500 m radius had the best fit (in bold).

**Model**	**K**	**Model Likelihood**	**AICc**	**ΔAICc**	**Weight**	**R^2**	**Adj. R^2**
**fT3 ~ Location + HMI + Season + Livestock density**	**11**	**-668.92**	**1360.32**	**0.00**	**0.54**	**0.22**	**0.21**
fT3 ~ Location + HMI + Season + Livestock density + NDVI	12	-668.89	1362.36	2.04	0.19	0.22	0.21
fT3 ~ Location + HMI^2 + Season + Livestock density + $NDVI^2$	12	-668.89	1362.36	2.04	0.19	0.22	0.21
fT3 ~ Location + HMI + Season + Livestock density + NDVI + Age group	14	-667.98	1364.74	4.42	0.06	0.22	0.21
fT3 ~ Location + HMI + Season	9	-675.03	1368.40	8.08	0.01	0.20	0.19
fT3 ~ Land use type + HMI + Season + Livestock + NDVI	10	-675.28	1370.97	10.65	0.00	0.20	0.19
fT3 ~ Land use type + HMI^2 + Season + Livestock density + $NDVI^2$	10	-675.28	1370.97	10.65	0.00	0.20	0.19
fT3 ~ Land use type + HMI + Season + Livestock density	9	-677.50	1373.33	13.01	0.00	0.20	0.19
fT3 ~ Land use type + HMI + Season + Livestock density + NDVI + Age group	12	-674.50	1373.57	13.25	0.00	0.21	0.19
fT3 ~ Land use type + HMI + Season	7	-687.03	1388.26	27.94	0.00	0.17	0.16
fT3 ~ Location + HMI	8	-697.30	1410.87	50.55	0.00	0.14	0.13
fT3 ~ Location	7	-699.02	1412.25	51.93	0.00	0.13	0.12
fT3 ~ Land use type + HMI	6	-706.30	1424.74	64.43	0.00	0.11	0.10
fT3 ~ Land use type	5	-712.09	1434.29	73.97	0.00	0.09	0.09
